# Lack of Effect of Gluten Challenge on Fecal Microbiome in Patients With Celiac Disease and Non-Celiac Gluten Sensitivity

**DOI:** 10.14309/ctg.0000000000000441

**Published:** 2021-12-20

**Authors:** Yael R. Nobel, Felix Rozenberg, Heekuk Park, Daniel E. Freedberg, Martin J. Blaser, Peter H.R. Green, Anne-Catrin Uhlemann, Benjamin Lebwohl

**Affiliations:** 1 Celiac Disease Center, Columbia University Irving Medical Center, New York, New York, USA;; 2 Microbiome and Pathogen Genomics Collaborative Center, Columbia University Irving Medical Center, New York, New York, USA;; 3 Center for Advanced Biotechnology and Medicine, Rutgers University, New Brunswick, New Jersey, USA;; 4 Department of Epidemiology, Mailman School of Public Health, Columbia University Irving Medical Center, New York, New York, USA;; 5 Division of Infectious Diseases, Department of Medicine, Columbia University Irving Medical Center, New York, New York, USA.

## Abstract

**INTRODUCTION:**

Celiac disease (CD) may be associated with gut microbial dysbiosis. Whether discrete gluten exposure in subjects with well-controlled disease on a gluten-free diet impacts the gut microbiome is unknown and may have implications for understanding disease activity and symptoms. We conducted a prospective study to evaluate the impact of gluten exposure on the gut microbiome in patients with CD and nonceliac gluten sensitivity (NCGS).

**METHODS:**

Subjects with CD (n = 9) and NCGS (n = 8) previously on a gluten-free diet were administered a 14-day gluten challenge (5 g of gluten per day) and compared with controls (n = 8) on a usual gluten-containing diet. Stool was collected for fecal microbiome analysis using 16S rRNA gene and metagenomic sequencing before, during, and after the gluten challenge. Symptoms were assessed using 2 validated clinical scales.

**RESULTS:**

Among subjects with CD and NCGS, there were no significant fecal microbial changes in response to gluten challenge. Gut microbiome composition differed among controls, subjects with CD, and subjects with NCGS at baseline, and these differences persisted despite gluten exposure. Gastrointestinal and general health symptoms reported by subjects with CD and NCGS were worst in the middle of gluten challenge and lessened by its end, with no consistent associations with gut microbiome composition.

**DISCUSSION:**

Pre-existing fecal microbiome diversity was unaffected by gluten challenge in adult subjects with CD and NCGS. These findings suggest that current microbiome status is unrelated to current disease activity and disease severity.

## INTRODUCTION

Celiac disease (CD) is a multisystem autoimmune disease triggered by ingestion of gluten in genetically susceptible individuals and is treated by avoidance of dietary gluten (1). Another condition, nonceliac gluten sensitivity (NCGS), describes patients who develop symptoms—such as diarrhea, abdominal pain, or nausea—in response to gluten intake, but do not carry serologic or histologic markers of CD (2). In patients with CD and NCGS observing a gluten-free diet, there is wide variation in symptoms and, in CD, degree of biomarker recurrence that develops in response to gluten exposure, ranging from negligible to severe.

Investigations of CD have posited a role of the gut microbiome in multiple aspects of disease development and progression (3-5). Certain taxa, including *Bifidobacterium* and Clostridial species such as *Faecalibacterium prausnitzii*, have decreased abundance in patients with active CD or treated CD compared with controls (6,7). However, not all studies have shown consistent trends, and some of the dysbiosis observed in patients with CD may be attributable to gluten-free diet rather than to the underlying disease (8,9).

In this study, we investigated the impact of gluten exposure on gut microbiome composition among a clinic-based cohort of patients with CD and NCGS compared with controls. We hypothesized that a 14-day gluten challenge would alter gut microbiome composition in subjects with CD and NCGS and that symptom severity in response to gluten challenge would be associated with microbiome composition.

## METHODS

**Figure 1.**
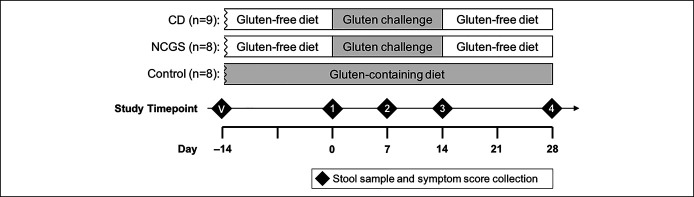
Study design. CD, celiac disease; NCGS, nonceliac gluten sensitivity; V, validation timepoint.

We performed a prospective study in which patients with CD and NCGS maintaining a long-term gluten-free diet were given a 14-day gluten challenge in parallel with controls, who remained on a nonrestrictive, gluten-containing diet (Figure 1). Stool sample collection for gut microbiome analysis and symptom assessments were performed before, during, and after the gluten challenge or corresponding period for the controls. We characterized baseline differences, stability over time, and response to gluten challenge of the gut microbiome and correlation of the gut microbiome with clinical symptoms in patients with CD and NCGS.

### Study design and subject recruitment

Adult subjects 18 years and older were included in the study. All subjects with CD (n = 9) and subjects with NCGS (n = 8) were recruited by their physicians at the Celiac Disease Center at Columbia University, New York, New York, and control subjects (n = 8) were recruited through fliers posted at Columbia University Irving Medical Center. Four other study participants with CD were enrolled but did not complete the study because of severe symptoms at the outset of gluten challenge (n = 2) or loss to follow-up (n = 2). CD diagnosis was based on intestinal biopsy performed as standard care before study screening. NCGS was defined by the presence of gastrointestinal and/or extraintestinal symptoms that improved on cessation of gluten ingestion, with negative genetic, serologic, or duodenal histologic testing for celiac disease while on a gluten-containing diet. Controls had no clinical history of gluten intolerance or intestinal disease. Exclusion criteria included a personal history of inflammatory bowel disease, hospitalization in the 2 months before study enrollment, history of severe symptomatic response to gluten exposure, or use of antibiotics, corticosteroids, or probiotics (at least once per week) in the 3 months before study enrollment. Baseline clinical characteristics were compared among the 3 study groups using ANOVA for continuous variables and the χ^2^ test or Fisher exact test for categorical variables.

The study duration was 6 weeks. This was composed of 3 phases for subjects in the CD and NCGS groups: a 2-week lead-in period of usual (gluten-free) diet; a 2-week period of gluten challenge, during which subjects consumed 5 g of gluten (2 slices of whole wheat bread, with gluten content confirmed through R5 ELISA by Bia Diagnostics [Colchester, VT], an accredited food and nutraceutical testing laboratory) per day; and a 2-week follow-up period of usual (gluten-free) diet. Controls continued their usual (gluten-containing) diets for the duration of the study. Clinical symptom questionnaires and stool samples were collected at 5 timepoints before, during, and after the gluten challenge.

This study was approved by the Columbia University Irving Medical Center Institutional Review Board.

### Symptom assessments and symptom group assignments

The Gastrointestinal Symptom Rating Scale (GSRS; AstraZeneca, East Hartford, CT) is a validated clinical rating scale for describing patient-reported gastrointestinal symptoms across 5 symptom dimensions, with higher score indicating more severe symptoms. The RAND 36-Item Health Survey 1.0 short-form survey (SF-36; RAND Corporation, Santa Monica, CA) is a validated questionnaire assessing patient-reported outcomes in 8 health domains, with lower score indicating more severe symptoms (10). At timepoints 2, 3, and 4, the total number of symptom dimensions (GSRS) or health domains (SF-36) indicating worsening, improving, or unchanged severity compared with timepoint 1 was assessed, with each of the symptom complexes weighted equally. Subjects were considered to have high (more severe) overall symptoms for a given timepoint if the number of worsened symptom complexes exceeded the number of symptom complexes that improved and/or were unchanged compared with baseline timepoint 1 and considered to have low overall symptoms otherwise.

Because diarrhea is a prominent symptom in clinically active CD and NCGS, the diarrhea component of the GSRS was also independently examined. For each subject, the diarrhea score at timepoint 2, 3, or 4 was compared with timepoint 1. Subjects were considered to have high (more severe) diarrhea symptoms for a given timepoint if the diarrhea score increased compared with baseline timepoint 1 and to have low diarrhea symptoms if the diarrhea score decreased or stayed the same.

Symptom severity within each study group at timepoints 2, 3, and 4 was compared with timepoint 1 using *t* tests with *P* value adjusted for multiple comparisons.

### Stool sample collection

Study subjects collected stool specimens at each of the prespecified timepoints. Stool was submitted within 24 hours of collection and stored as unprocessed aliquots at –80^o^C.

### 16S rRNA sequencing and analysis

DNA was extracted from thawed stool samples using the Qiagen MagAttract PowerSoil Kit. The V3-V4 region of the 16S rRNA gene was polymerase chain reaction amplified using standard primers with overhang for Nextera XT index adapters (Illumina, San Diego CA) (11). Final libraries were quantitated using Quant-iT Broad Range dsDNA Assay Kit (Thermo Fisher Scientific, Waltham, MA) and were sequenced on the MiSeq platform to achieve 300-bp paired-ended reads, yielding an average of 37,890 reads per sample. Analysis was performed using Divisive Amplicon Denoising Algorithm version 2 (DADA2) v1.10.1 and R version 3.6.1 (12). DADA2 was used for quality filtering, trimming, error correction, chimeric sequence removal, and generation of the amplicon sequence variant (ASV) table. Each ASV was classified using the Greengenes 97% reference database. ASVs composing on average less than 0.05% of relative abundance were removed. Alpha diversity (Shannon index) and beta diversity (unweighted UniFrac, weighted UniFrac, and Bray-Curtis) were determined using the phyloseq v1.30.0 package in R (13). Differences in alpha diversity were compared across disease group and across timepoints using a pairwise Kruskal-Wallis test. Beta diversity was visualized using principal coordinate analysis (PCoA), with differences tested using permutational analysis of variance (PERMONVA) using the R package, vegan v2.5.6 (14). Differentially abundant taxa were identified using DESeq2 after false discovery rate adjustment for multiple comparisons of the *P* values (15). Bacterial DNA sequences are publicly available in the NCBI Sequencing Read Archive (SRA) (Accession number PRJNA778253).

### Shallow shotgun metagenomic sequencing and analysis

The same extracted DNA was used to create shotgun metagenomics libraries on select samples using the Nextera DNA Flex Library Prep kit (Illumina) according to the standard protocol while using the minimum number of cycles for polymerase chain reaction amplification. Samples from subjects with CD and NCGS at timepoints 1 and 3, immediately before and after gluten challenge, were selected because these were expected to best demonstrate changes to the gut microbiome in response to gluten exposure. Libraries were quantitated using Quant-iT Broad Range dsDNA Assay Kit (Thermo Fisher Scientific), and fragment length was validated using the 2,100 Bioanalyzer system (Agilent, Santa Clara, CA) with a High-Sensitivity DNA Chip. Sequencing was performed on the NextSeq platform to generate 150-bp single-ended reads. Raw reads were filtered to remove low-quality reads and adapters using Trim Galore version 0.6.4. After quality control filtering, the average read count per sample was approximately 4.4 million reads with no samples reaching below 2.5 million reads. Use of shotgun metagenomics sequenced at a shallow read depth, beginning with 0.5 million reads, has recently been validated for beginning to investigate species-level taxonomic and functional microbiome data (16). Kraken2 version 2.0.8 was used for taxonomic classification using the available bacterial genome information available on the National Center for Biotechnology Information (17). The taxonomic read count table was normalized using reads per million raw reads (RPKM) to control for bias introduced by variable genome size. RPKM was then used to determine the relative abundance of each taxon. Functional analysis of gene and pathway abundance was performed using HUMAnN2 version 2.8.1 with default settings using the UniRef 50 database (18). Identified functional pathway differences and the associated taxonomic drivers were presented using Functional Shifts Taxonomic Contributors (FishTaco) analysis version 1.1.1 (19).

## RESULTS

### Baseline clinical characteristics and microbiome composition of study subjects

Baseline clinical characteristics of the study population are shown in Table 1. There were no significant differences in demographics among the 3 groups or in previous dietary exposures between the CD and NCGS groups. Study design is shown in Figure 1.

**Table 1.** Characteristics of study population (N = 25 subjects)

	Control (n = 8)	NCGS (n = 8)	CD (n = 9)	*P* value
Age at start of study (y), median (IQR)	40.4 (34.5–51.4)	51.9 (40.7–76.5)	57.9 (48.8–70.6)	0.07
Sex, no. (%)				
Female	4 (50.0)	8 (100.0)	5 (55.6)	1.0^b^
Male	4 (50.0)	0 (0.0)	4 (44.4)	
Duration of GFD before study start (y), median (IQR)	N/A	3.2 (1.2–9.1)	8.4 (3.6–13.0)	0.15
Time since last suspected inadvertent gluten exposure at study start (d), median (IQR)^a^	N/A	37 (6–84)	9 (1–48)	1.0

CD, celiac disease; GFD, gluten-free diet; IQR, interquartile range; NCGS, nonceliac gluten sensitivity.

a Data missing for 1 patient with CD.

b Comparing only CD with NCGS: *P* = 0.08.

**Figure 2.**
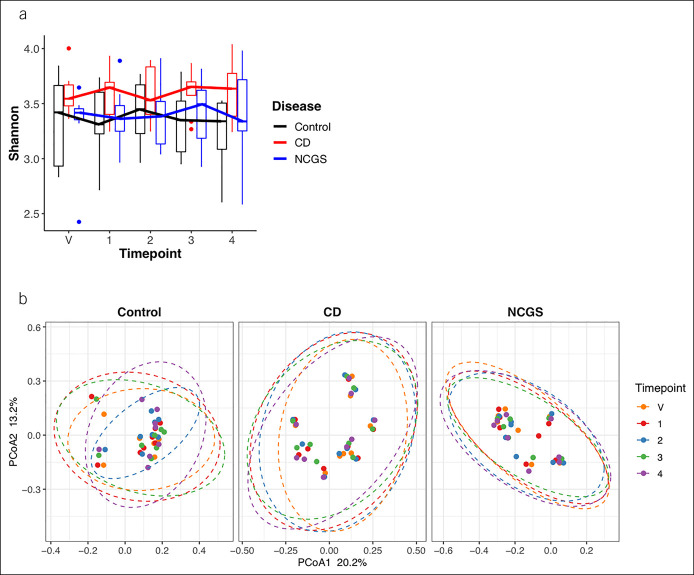
(**a**) Alpha diversity in control, CD, and NCGS study groups over time. (**b**) Beta diversity based on unweighted UniFrac within groups over time. All pairwise comparisons of timepoints within each study group: not significant at alpha = 5%. CD, celiac disease; NCGS, nonceliac gluten sensitivity.

To assess baseline consistency of the gut microbiome composition within each group, samples were collected at a validation timepoint, 14 days before timepoint 1. Between the validation timepoint and timepoint 1, microbiome composition was stable within each study group, with few differences in relative abundance of specific taxa and no differences in alpha or beta diversity (Figure 2).

### Gut microbiome composition in response to gluten challenge

There were no significant changes in gut microbiome composition during or after gluten challenge in either the CD or NCGS group. Between start and end of gluten challenge (timepoint 1 vs timepoint 3), there were no differences in alpha diversity, beta diversity, or differential abundance of specific taxa within either group (Figure 2; see supplementary Figure 1A, Supplementary Digital Content 1A, http://links.lww.com/CTG/A734, see supplementary Figure 2A , Supplementary Digital Content 2A, http://links.lww.com/CTG/A734).

**Figure 3.**
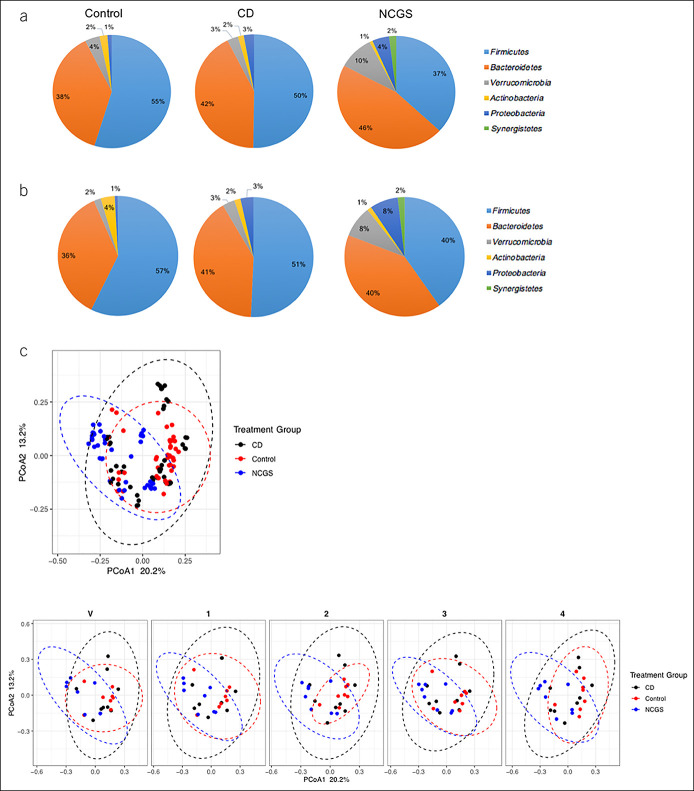
Microbiome composition in control, CD, and NCGS study groups over time. Relative abundance of phyla at (**a**) timepoint 1 and (**b**) timepoint 3. All other phyla had relative abundance <0.5%. (**c**) Unweighted UniFrac within each group and in the 3 treatment groups over time. Pairwise comparisons between each group, *P* < 0.01. CD, celiac disease; NCGS, nonceliac gluten sensitivity.

Baseline gut microbiome composition differed among the 3 study groups, and these differences persisted after gluten challenge (Figure 3A). Although alpha diversity did not differ among the 3 study groups over time (Figure 2A), beta diversity was significantly different among the 3 study groups at all timepoints (Figure 2B; see supplementary Figure 1B, Supplementary Digital Content 1B, http://links.lww.com/CTG/A734, see supplementary Figure 2B, Supplementary Digital Content 2B, http://links.lww.com/CTG/A734). Based on 16S rRNA analyses, multiple specific taxa were differentially abundant in CD and NCGS groups compared with controls at timepoints 1, 2, 3, and 4 (see supplementary Figures 4-6, Supplementary Digital Content 4-6, http://links.lww.com/CTG/A734). *Akkermansia muciniphila* was enriched in subjects with CD compared with the control group at all timepoints and in NCGS compared with the control group at timepoint 2 only.

### Shotgun metagenomics and functional analyses

Shotgun metagenomic sequencing was performed on CD and NCGS groups at baseline (timepoint 1) and end of gluten challenge (timepoint 3). Using this method, there remained no significant differences in alpha diversity, beta diversity, or differential abundance of any specific taxon between the 2 timepoints within either group. The metagenome-based and taxa-based functional capacity of the microbiome was evaluated using Functional Shifts Taxonomic Contributors (FishTaco) analysis (19); comparing timepoints 1 and 3, there were no significant differences in functional pathways within either group.

### Association of gut microbiome composition with clinical outcomes after gluten challenge

The CD and NCGS groups had more severe symptoms than controls at all timepoints (see supplementary Figures 7 and 8, Supplementary Digital Content 7 and 8, http://links.lww.com/CTG/A734), with nonsignificant trend toward worsening of some symptoms over the course of the study. One question in the GSRS specifically evaluated nausea and vomiting; this symptom was most severe in the NCGS group throughout the study, but rose in subjects with CD after gluten exposure. The number of patients with high (more severe) or low (less severe) composite overall symptoms during and after gluten challenge relative to baseline is shown in supplementary Figure 9, Supplementary Digital Content 9, http://links.lww.com/CTG/A734.

Gut microbiome composition was evaluated within CD and NCGS study groups comparing subjects with high vs low overall symptoms at the end of gluten challenge, timepoints 3 and 4. In both groups, there were no significant differences in alpha or beta diversity at either timepoint between those with high and low overall symptoms. Differentially abundant taxa at each timepoint in the CD and NCGS groups are shown in supplementary Figure 10, Supplementary Digital Content 10, http://links.lww.com/CTG/A734. In both subjects with CD and NCGS, *Akkermansia muciniphila* was enriched in subjects with low overall symptoms at the end of gluten challenge (timepoint 3), but in subjects with high overall symptoms 2 weeks later (timepoint 4). In the CD group, *Faecalibacterium prausnitzii* was enriched at both timepoints after gluten challenge.

When comparing subjects with high vs low diarrhea symptoms after gluten challenge, there were no differences in alpha or beta diversity within either the CD or NCGS group. Differentially abundant specific taxa based on high or low diarrhea symptom in the CD and NCGS groups are shown in supplementary Figure 11, Supplementary Digital Content 11, http://links.lww.com/CTG/A734.

## DISCUSSION

In this prospective study of subjects with CD and NCGS undergoing a 14-day gluten challenge, we found that baseline fecal microbiome composition differed among subjects with CD, subjects with NCGS, and controls, but that within the CD and NCGS groups, gluten challenge led to no significant changes in gut microbiome composition or functional capacity over time. In addition, we found no differences in alpha and beta diversity between subjects with more vs less severe symptoms after gluten challenge and inconsistent differences in differential abundance of specific taxa. These findings provide strong evidence that periodic gluten exposure does not meaningfully disrupt the gut microbiome in people with CD and NCGS observing a gluten-free diet.

Previous studies have posited a role of the gut microbiome in the development and clinical manifestations of CD. In infancy, events that perturb the gut microbiome—including enteric infections and systemic antibiotic exposure—are associated with CD later in life (20-23). Relative abundance of *Bifidobacterium* and *F. prausnitzii*, a Clostridial species believed to serve an anti-inflammatory role in the intestine, has been found to be lower in children with CD than controls (6,7,24). Although it is likely that gut microbiome composition in childhood contributes to the pathogenesis of CD through its role in gut mucosal and systemic immune system development, the impact of gut dysbiosis later in life is less clear. Studies have suggested that adults with endoscopically, serologically, or symptomatically active CD may have higher relative abundance of *Proteobacteria* and lower relative abundance of *Firmicutes* in duodenal samples; however, consistent patterns of dysbiosis and the impact of gluten exposure or avoidance remain incompletely understood (5,24,25).

Long-term diet is a potent modulator of the gut microbiome and may contribute to a variety of pathology, ranging from metabolic dysregulation to colon cancer (26-30). In healthy controls, gluten-free diet is associated with reductions in *Bifidobacterium* abundance, changes in abundance of Clostridial species, and changes in metabolic pathways related to carbohydrate metabolism, mirroring changes observed in patients with CD (31-33). We found, however, that gluten challenge in patients with established CD or NCGS produced few changes in fecal microbial diversity or relative abundance of specific taxa. Subjects with CD and NCGS had increased relative abundance of *Bacteroidetes* and *Proteobacteria* and decreased relative abundance of *Firmicutes*; these differences were present at the start of the study and persisted despite gluten intake. Although composition of the microbiome does not always reflect its metabolic function, we also found no changes in functional capacity of the microbiome after gluten challenge.

We investigated the possibility that changes in gut microbiome composition mediate variable symptomatic response to gluten ingestion among patients with CD and NCGS. Both within our study over time and in our study compared with previous studies, enrichment of specific taxa correlating to specific symptoms was variable. In our study, *Bifidobacterium* was enriched in both subjects with CD and NCGS with more severe diarrhea, and *F. prausnitzii* was enriched in subjects with CD with more severe overall symptoms. *Akkermansia muciniphila* was enriched in both subjects with CD and NCGS with less severe overall symptoms at the end of gluten challenge, but in subjects with more severe symptoms by 2 weeks later. Some of our findings (e.g., the association of diarrhea with reduced abundance of *A. muciniphila*) have been identified previously (34), whereas others contradict some previous findings (e.g., that endoscopically active CD was associated with reduced abundance of *Bifidobacterium* and *F. prausnitzii)* (6). Based on the lack of change in microbial diversity during gluten challenge observed in our study, it seems that microbiome composition in adults with established CD is unlikely to contribute to symptom control, and interventions targeting the gut microbiome at this point in the disease process may have limited utility.

Our study has limitations. There was a small sample size in each study group, so it is possible that there was inadequate power to detect more subtle differences in gut microbiome composition. Symptom variability was limited, and patients with CD and NCGS with more severe baseline symptoms may be less likely to participate in a gluten challenge study. Because this study did not include children or adolescents, our null findings regarding microbial diversity may not necessarily apply across the lifespan. Longitudinal microbiome analysis was not possible for the 4 subjects with CD who left the study, including 2 who left because of symptoms; we were therefore unable to investigate associations between gut microbiome composition and CD in those with the most severe response to gluten. We studied the fecal microbiome, which is removed from the primary site of disease in CD; it is possible that duodenal dysbiosis would develop more quickly in response to gluten exposure. Future studies should consider endoscopic evaluation for duodenal microbial sampling after gluten challenge.

There were also several strengths. The patients were prospectively recruited and well-characterized, including biopsy-proven disease in subjects with CD. Because of the potential for significant symptoms including nausea, pain, and diarrhea, patients with CD and NCGS are often reluctant to participate in research requiring intentional repeated gluten exposure; our study is valuable in that it provides longitudinal analysis of the gut microbiome in these patients during and after gluten exposure. This important information is often difficult to obtain, and the analysis of serially collected fecal microbiome samples during a gluten challenge as it correlates with symptoms has not been evaluated by other studies. Subjects were sampled serially over time, allowing for prospective evaluation of changes to the gut microbiome before, during, and after gluten challenge. The dose of gluten (5 g per day) administered was standardized, and the duration of exposure used in our study has been demonstrated to be adequate for induction of histologic and serologic changes in patients with CD (35) and approach that of average daily gluten intake among US adults (36).

In conclusion, in this study of subjects with CD and NCGS previously on a long-term gluten-free diet, we found that a 14-day gluten challenge resulted in no significant changes to fecal microbiome composition in either group. Controls, subjects with CD, and subjects with NCGS had distinct microbiome composition at baseline, and differences persisted regardless of gluten exposure. When patients were stratified based on more severe or less severe symptoms in response to gluten challenge, there were no consistent patterns in gut microbial dysbiosis. Our findings indicate that in people with CD and NCGS on a long-term gluten-free diet, short-term gluten consumption does not alter gut microbiome composition. Gut microbial dysbiosis in patients with established CD or NCGS is unlikely to meaningfully impact disease activity and symptom severity in patients with these conditions.

## CONFLICTS OF INTEREST

**Guarantor of the article:** Benjamin Lebwohl, MD, MS.

**Specific author contributions:** Study concept and design: B.L., Y.R.N., M.J.B., P.H.R.G., A.C.U., and D.E.F. Data acquisition and analysis: Y.R.N., F.R., H.P., A.C.U., and B.L. Manuscript preparation: Y.R.N. and B.L. Manuscript review: Y.R.N., F.R., H.P., D.E.F., M.J.B., P.H.R.G., A.C.U., and B.L.

**Financial support:** Y.R.N.: NIH 5T32DK083256-12. D.E.F.: Columbia University Irving Institute for Clinical and Translational Research, and Department of Defense Peer-Reviewed Medical Research Program (PR181960). M.J.B.: Sergei S. Zlinkoff Fund for Medical Research & Education, Inc.; Canadian Institute of Advanced Research. ACU: R01 AI116939. B.L.: The Louis V. Gerstner, Jr. Scholars Program, the Celiac Disease Foundation Young Investigator Award, and the Louis and Gloria Flanzer Philanthropic Trust.

**Potential competing interests:** None to report.

**Study Highlights**

**WHAT IS KNOWN**

✓ Gut dysbiosis early in life may contribute to celiac disease (CD) pathogenesis.
✓ Whether gluten exposure in adults with established CD or nonceliac gluten sensitivity (NCGS) perturbs the gut microbiome is unknown.

**WHAT IS NEW HERE**

✓ In this gluten challenge study, fecal microbiome composition differed across study groups, but was not significantly altered by gluten exposure in the CD and NCGS groups.

## Supplementary Material

SUPPLEMENTARY MATERIAL
