# Supplementary material for: Lack of Effect of Gluten Challenge on Fecal Microbiome in Patients With Celiac Disease and Non-Celiac Gluten Sensitivity
Source: Clin Transl Gastroenterol. 2021 Dec 20;12(12):e00441. doi: 10.14309/ctg.0000000000000441 (PMC8691493; doi:10.14309/ctg.0000000000000441)

Supplementary Figure 1. (A) Weighted Unifrac within each group. All pairwise comparisons of timepoints within each study group: not significant at alpha=5%. (B) Weighted Unifrac of all groups over time. Pairwise comparisons between study groups: CD vs. control, p=0.03; NCGS vs. Control, p=0.006; CD vs. NCGS, p=0.01.

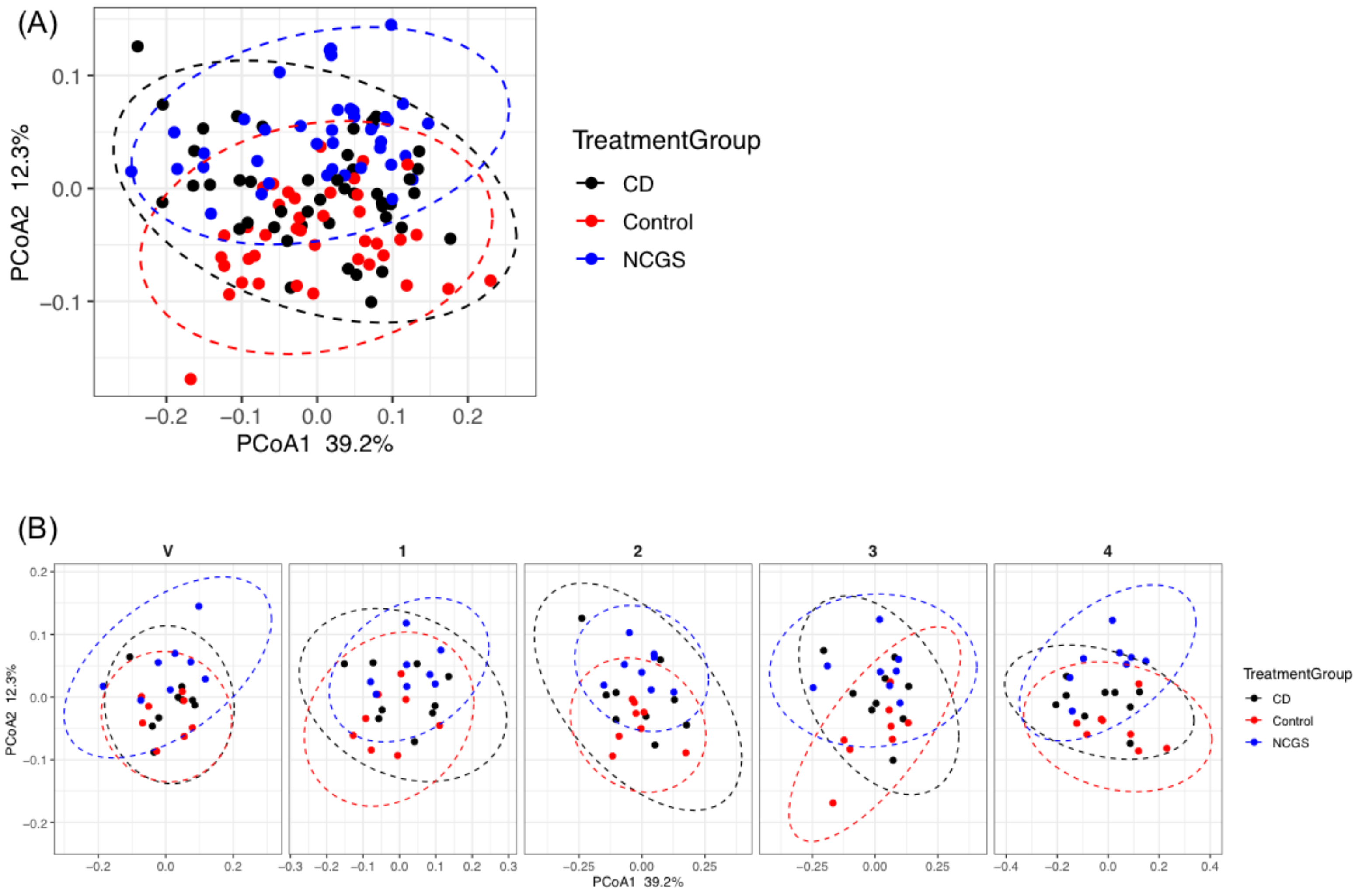

Supplementary Figure 2. (A) Bray-Curtis differences within each group. All pairwise comparisons of timepoints within each study group: not significant at alpha=5%. (B) Bray-Curtis distances of all groups over time. All study group comparisons p<0.001, adjusted for multiple timepoints.

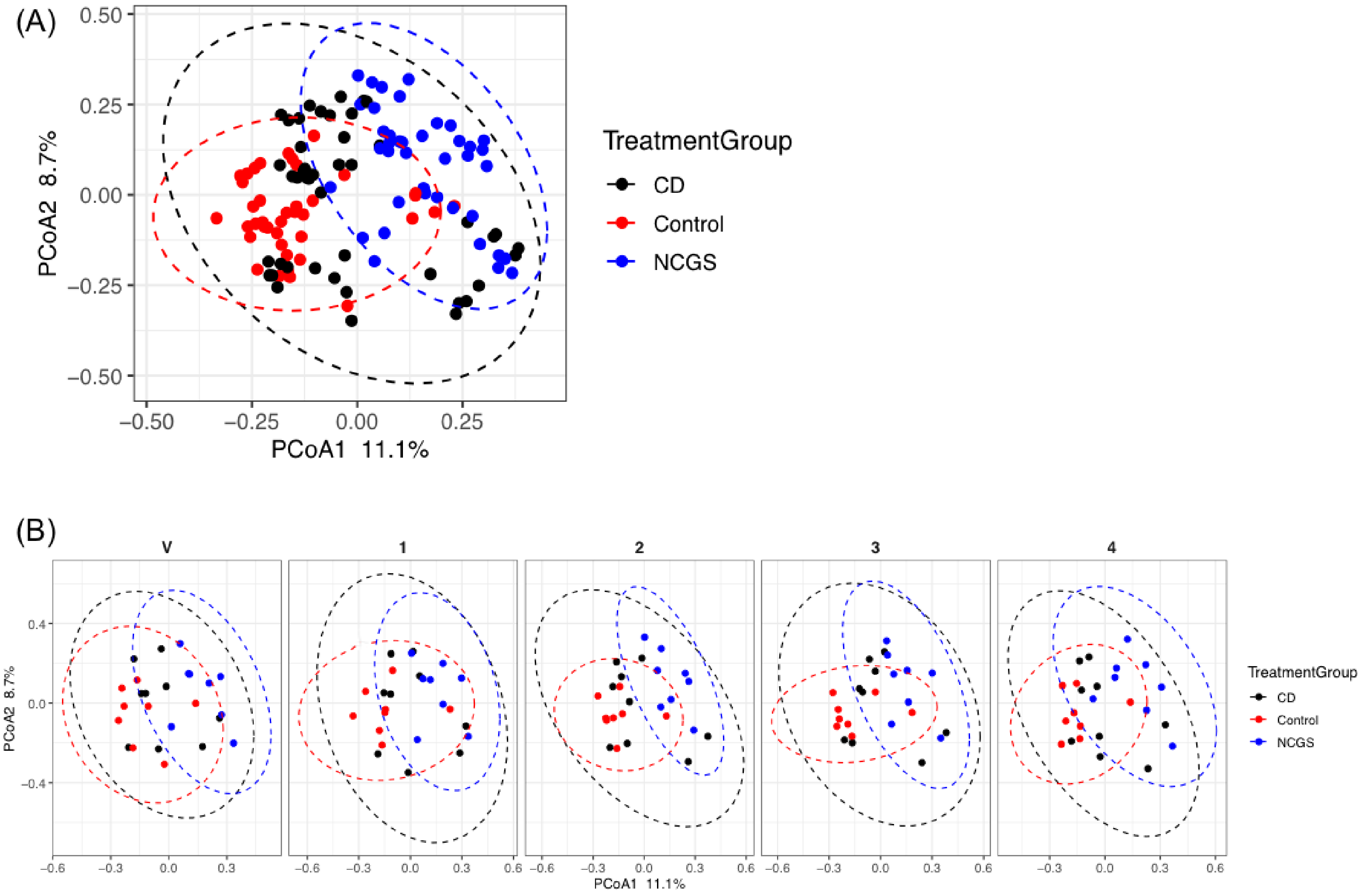

Supplementary Figure 3. Differential abundance of taxa at Timepoint 1 in CD and NCGS groups compared to control.

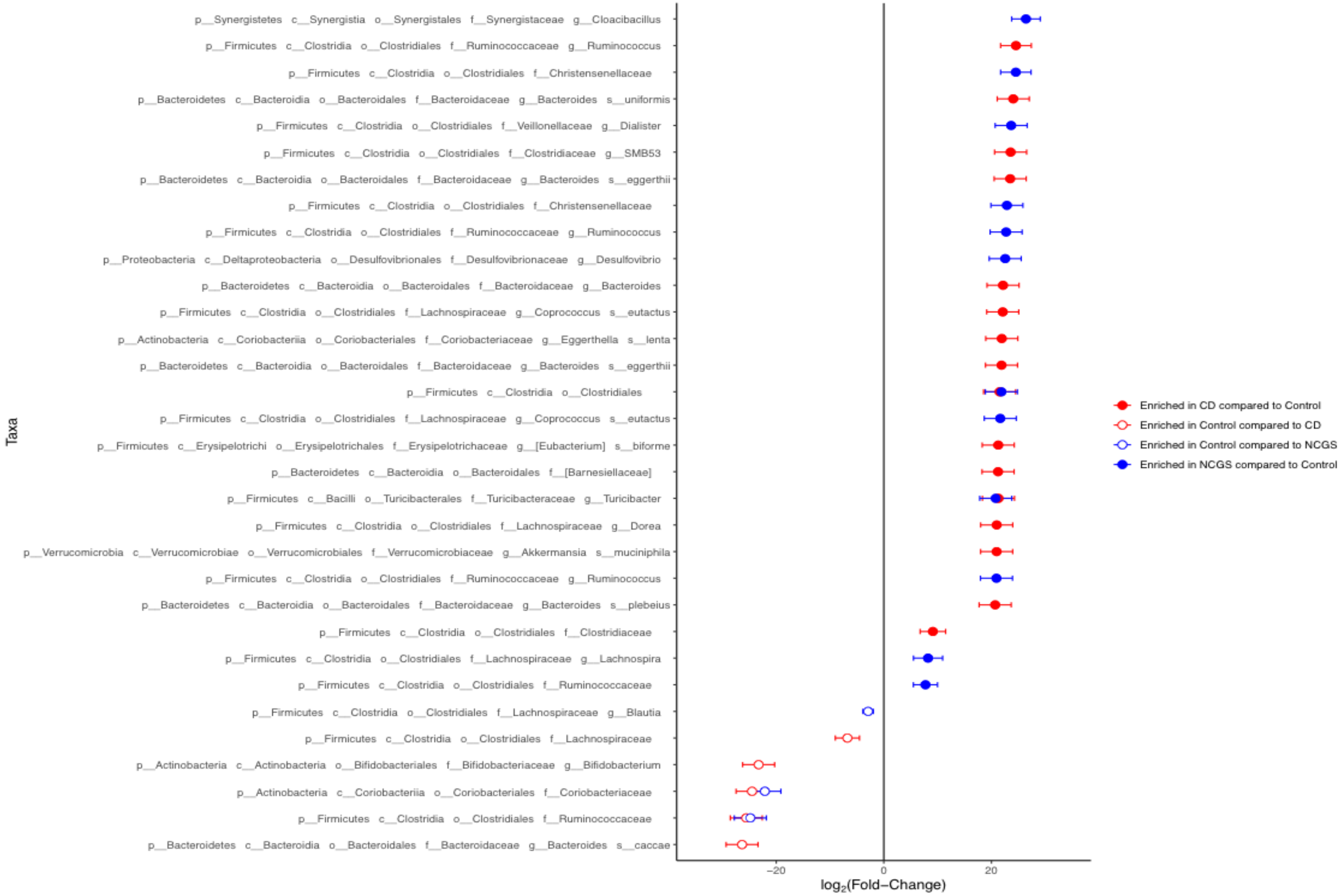

Supplementary Figure 4. Differential abundance of taxa at Timepoint 2 in CD and NCGS groups compared to control.

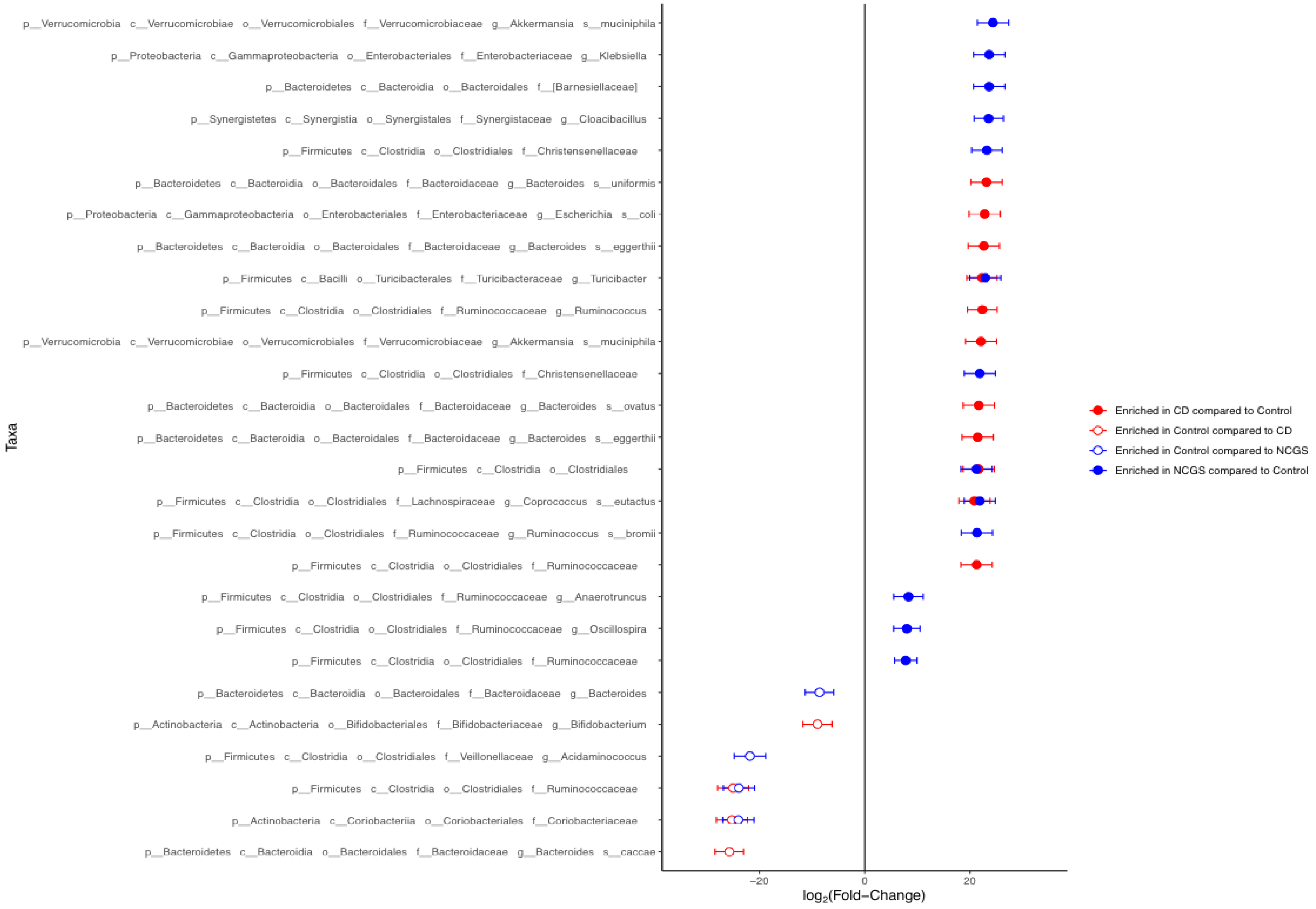

Supplementary Figure 5. Differential abundance of taxa at Timepoint 3 in CD and NCGS groups compared to control.

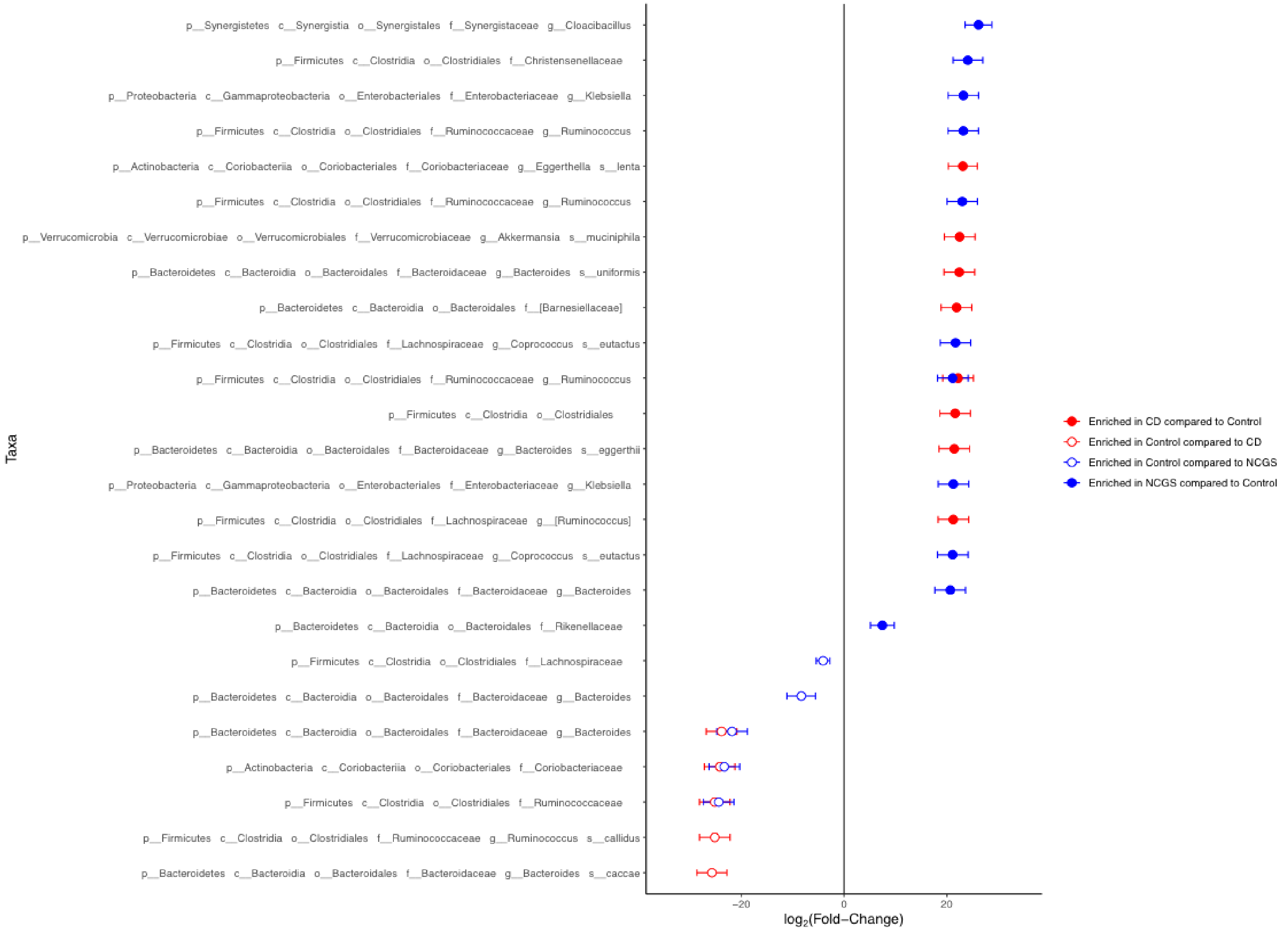

Supplementary Figure 6. Differential abundance of taxa at Timepoint 4 in CD and NCGS groups compared to control.

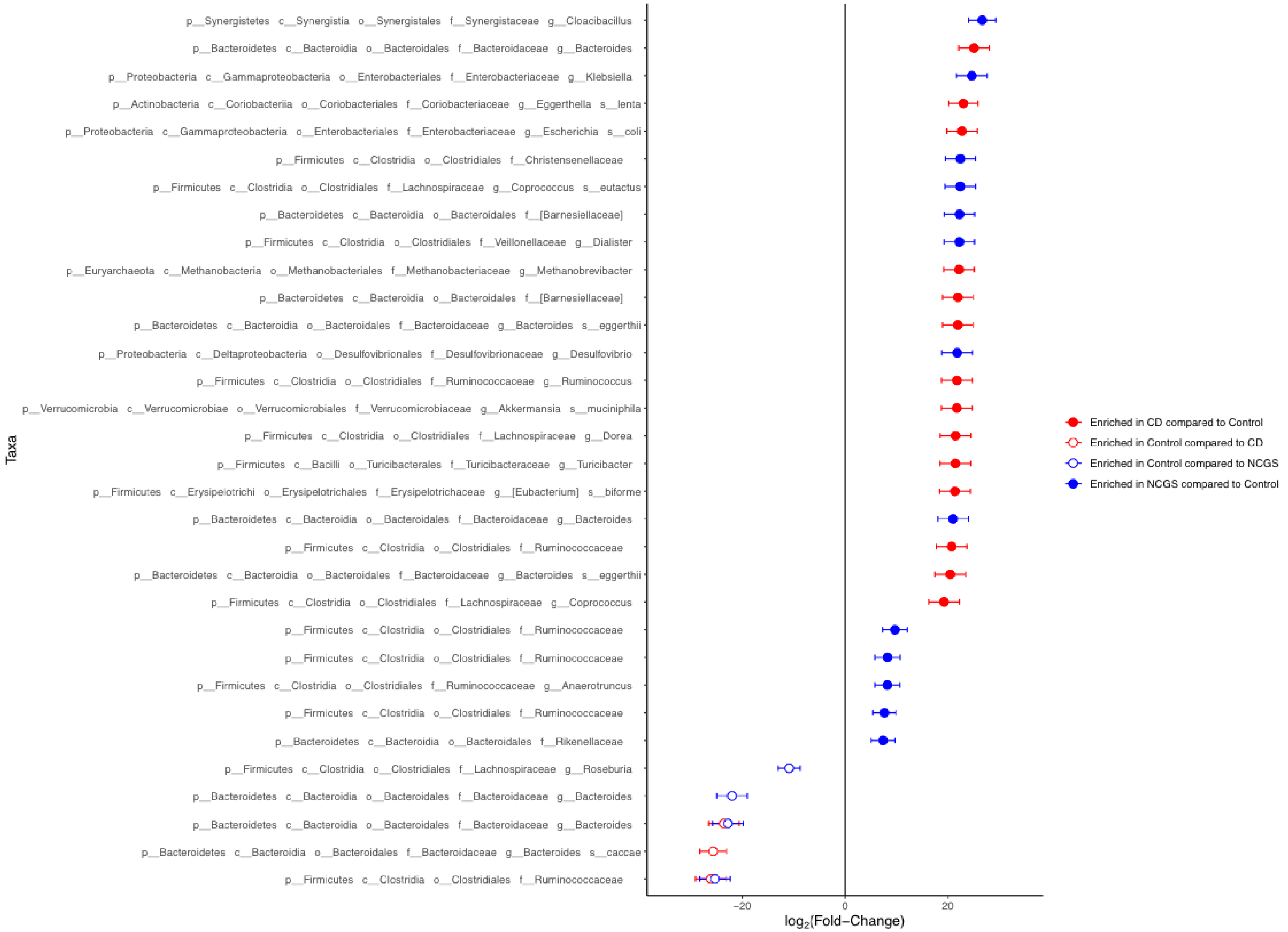

Supplementary Figure 7. (A) GSRS overall score and symptom subgroup scores for each study group. (B) Question 5 from the GSRS, which asks about nausea and vomiting. Scale 1-7, where higher score indicates more severe symptoms. All pairwise comparisons between Timepoint 2, 3, or 4 and Timepoint 1 within each study group: not significant at p<0.02, adjusting for multiple comparisons.

(A)

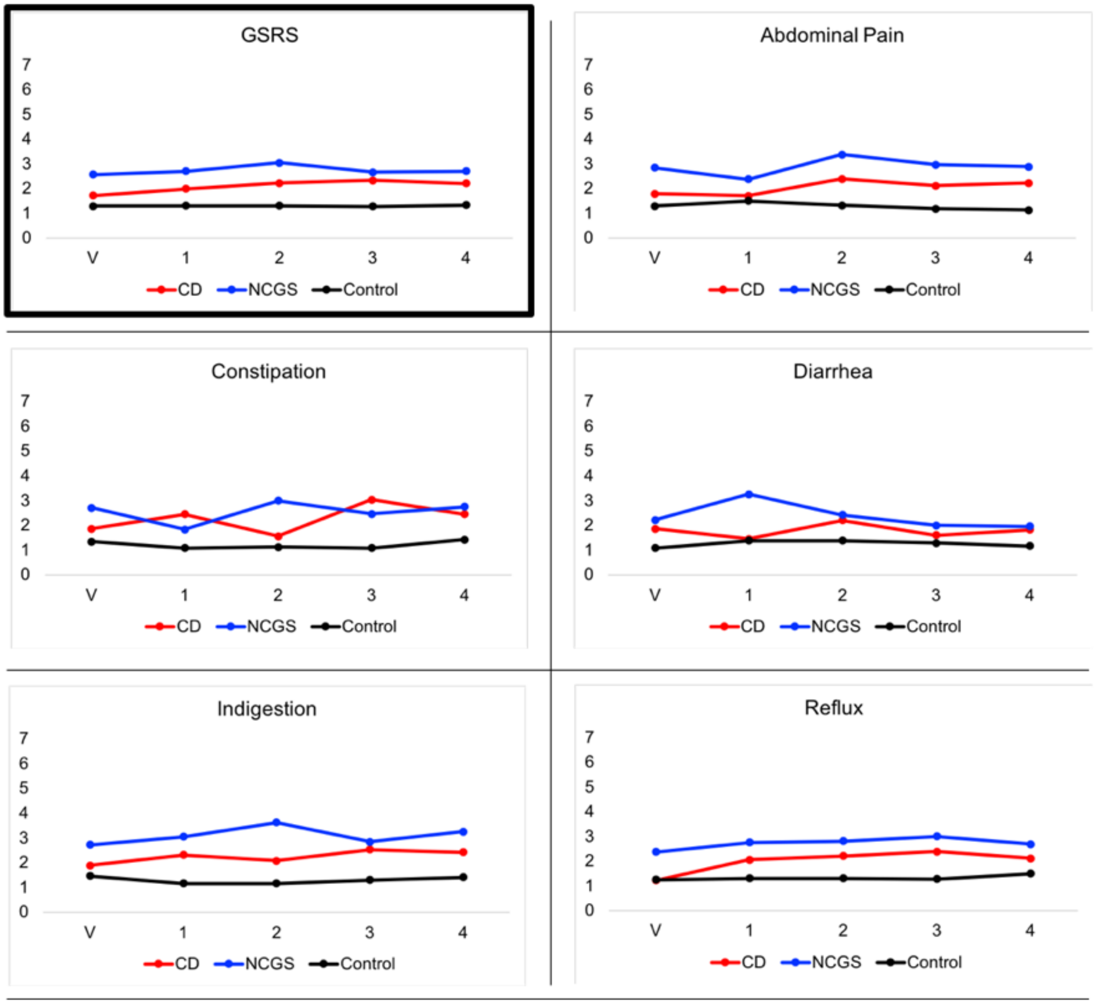

(B)

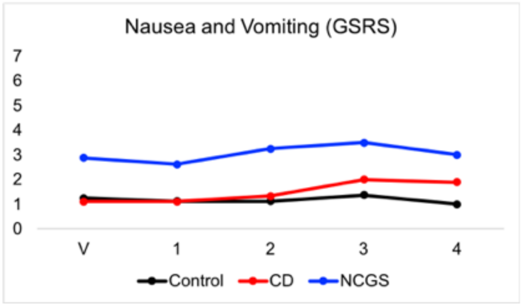

Supplementary Figure 8. SF36 health concepts. Each health concept is graded from 0-100% indicating percent of total possible score achieved, where higher score indicates more favorable health state and lower score indicates more severe symptoms. All pairwise comparisons between Timepoint 2, 3, or 4 and Timepoint 1 within each study group: not significant at $p<0.02$, adjusting for multiple comparisons.

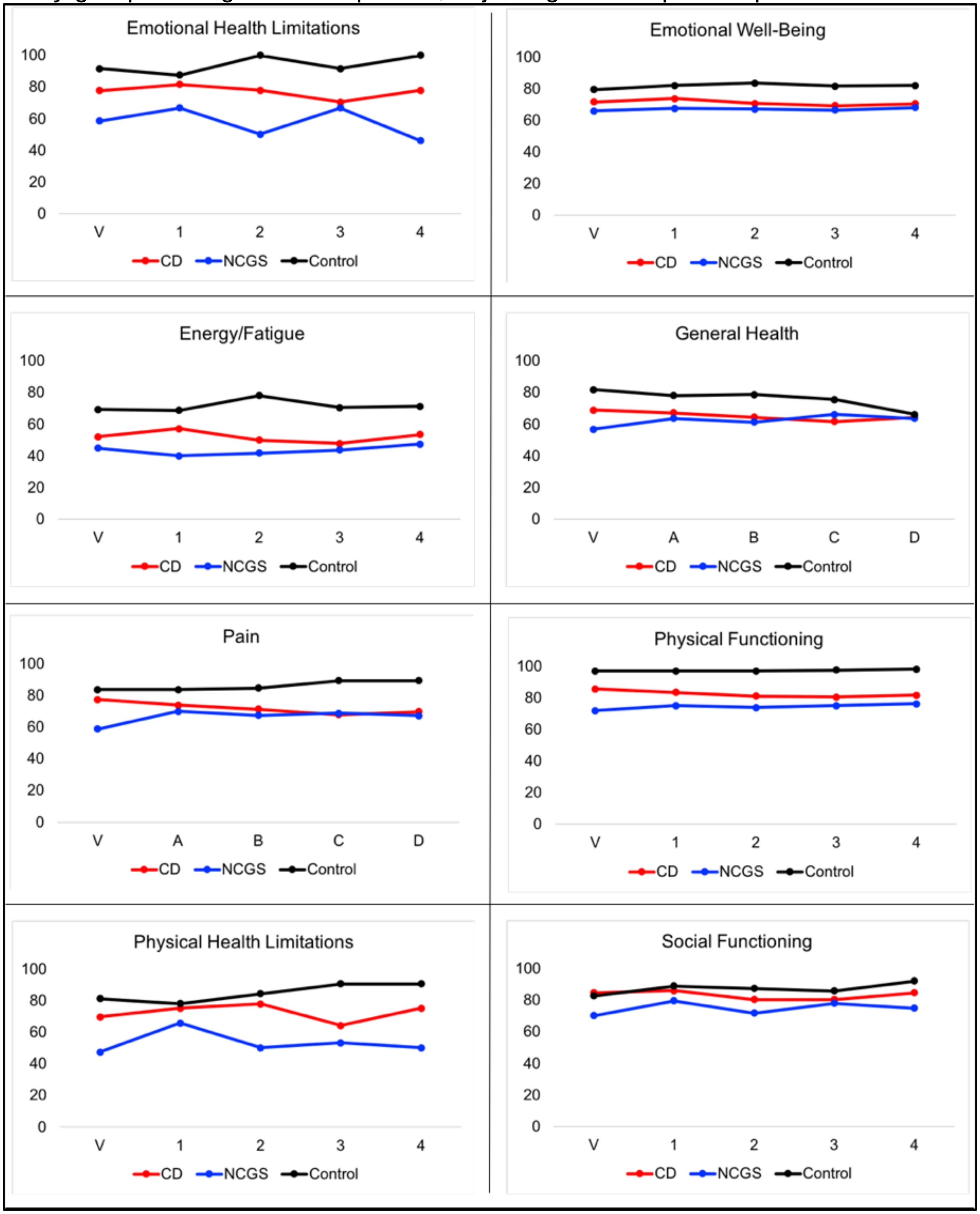

Supplementary Figure 9. (A) Numbers of subjects with high and low overall symptoms at Timepoints 2, 3, and 4 relative to Timepoint 1, according to treatment group. (B) Numbers of subjects with high and low diarrhea symptom at Timepoints 2, 3, and 4 relative to Timepoint 1, according to treatment group.

(A)

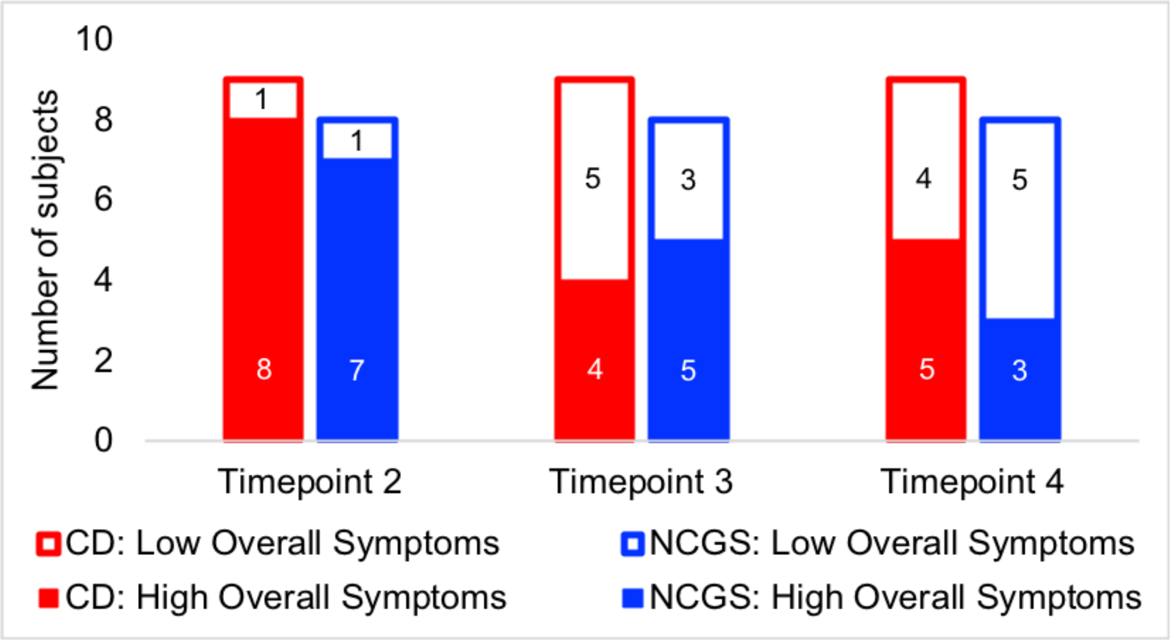

(B)

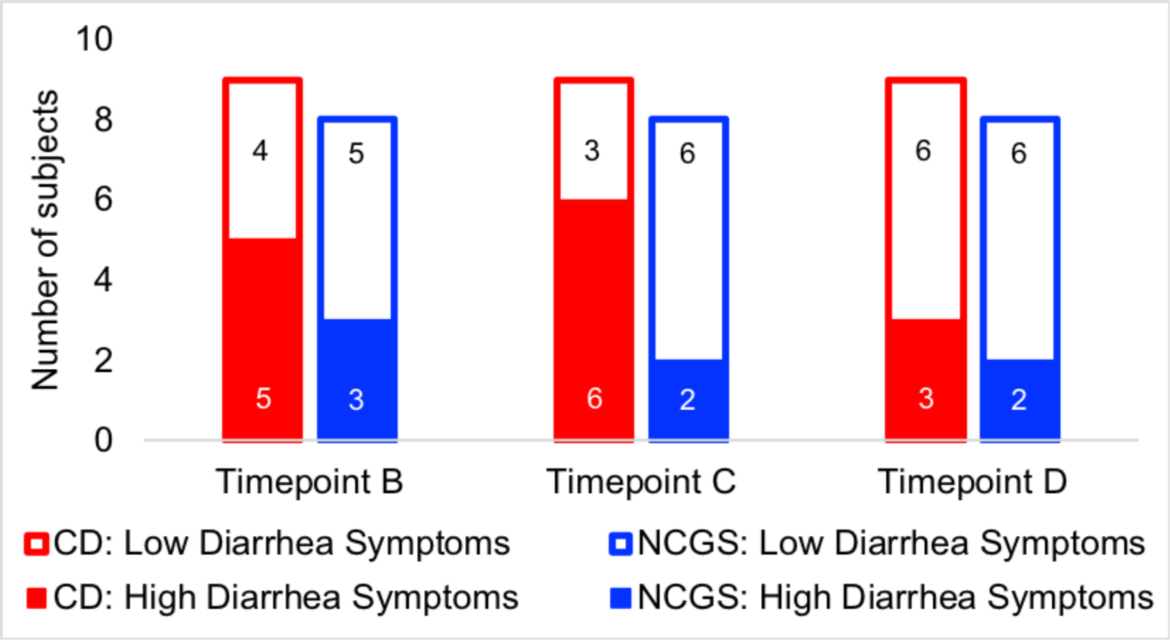

Supplementary Figure 10. Differential abundance of taxa within CD and NCGS groups in patients with high overall symptoms versus low overall symptoms at (A) Timepoint 3 and (B) Timepoint 4. *P*<0.05.

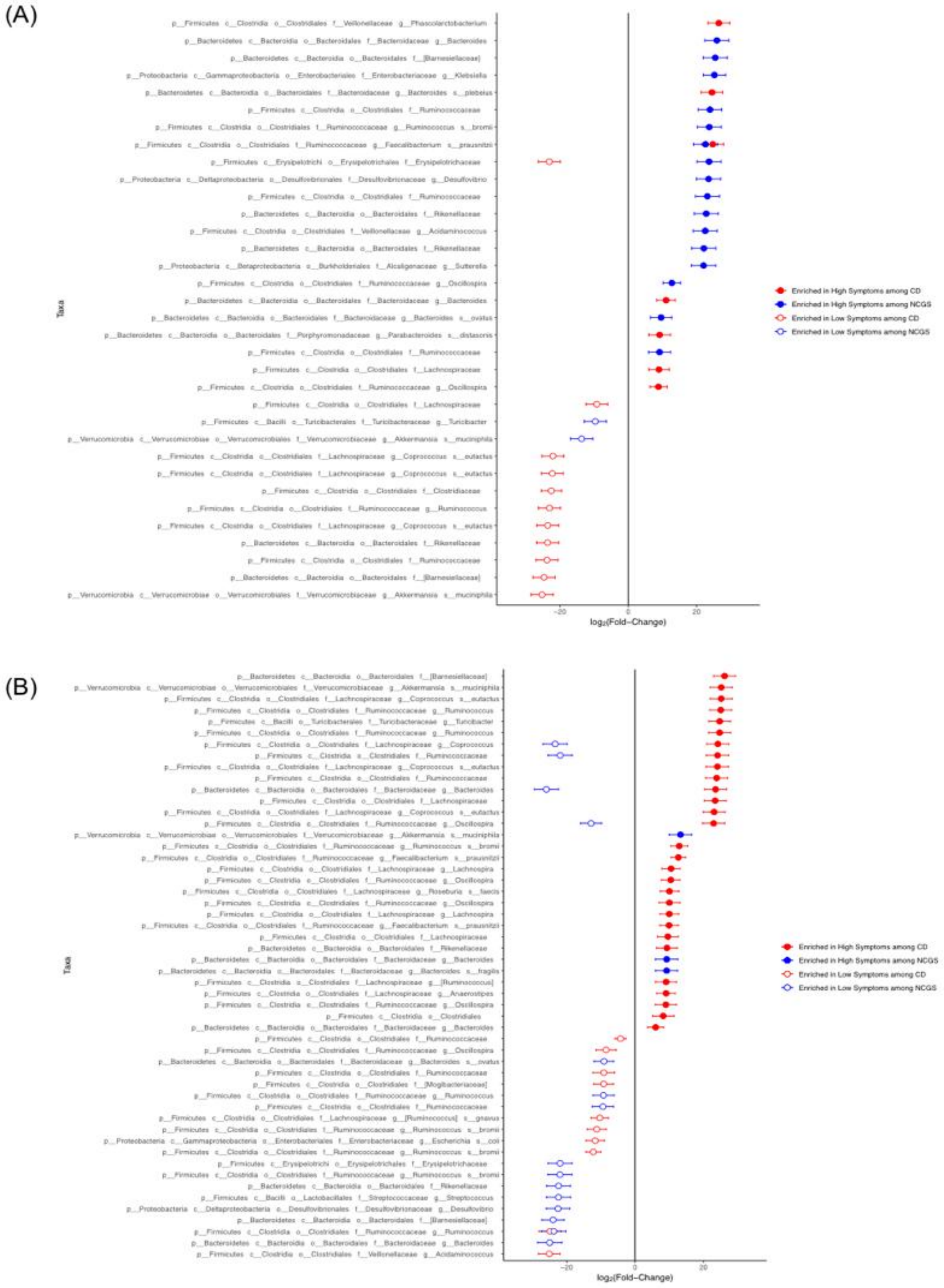

Supplementary Figure 11. Differential abundance of taxa within CD and NCGS groups in patients with high diarrhea symptom versus low diarrhea symptom at (A) Timepoint 3 and (B) Timepoint 4. *P*<0.05.

(A)

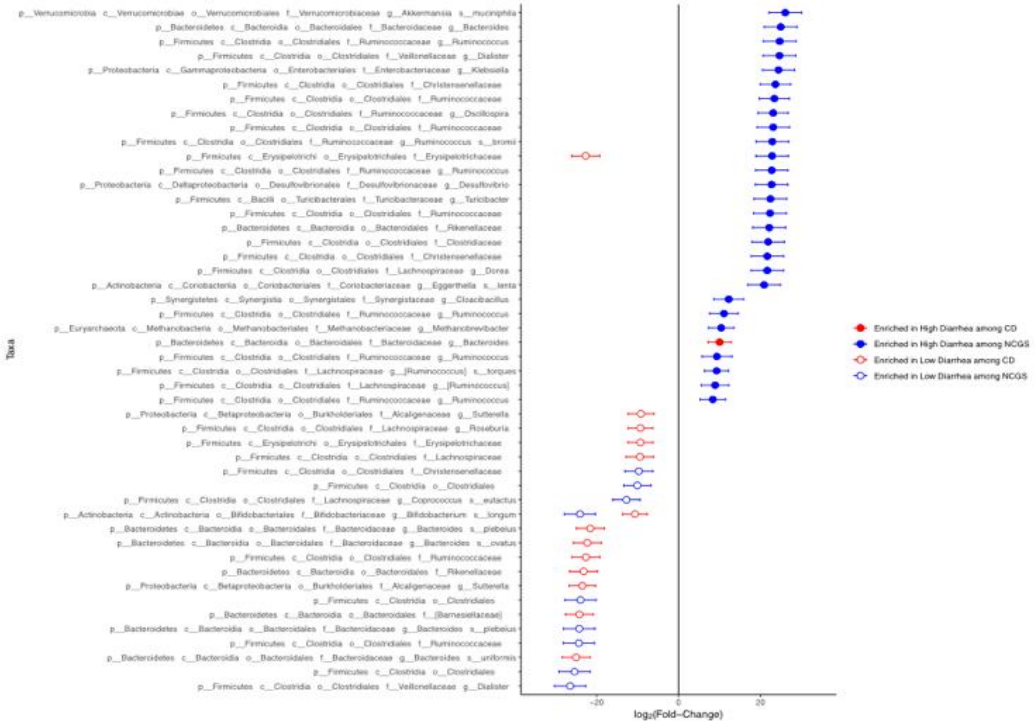

(B)

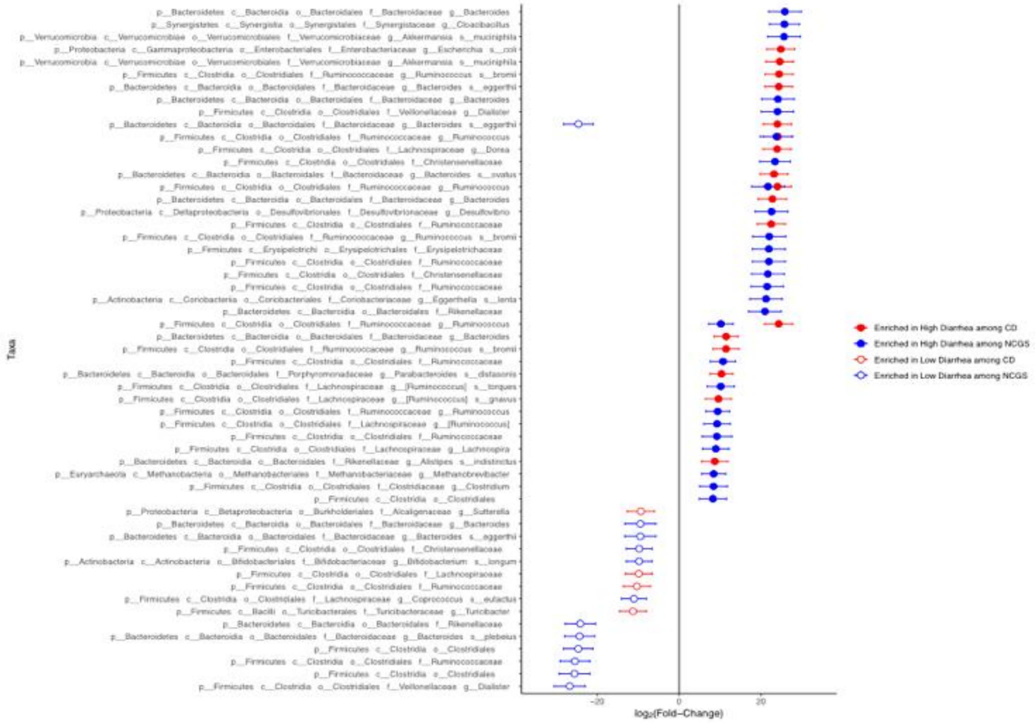

Supplement: SUPPLEMENTARY MATERIAL [file ct9-12-e00441-s001.pdf]
